# Interleukin‐18 in patients with acute coronary syndromes

**DOI:** 10.1002/clc.23274

**Published:** 2019-10-09

**Authors:** Axel Åkerblom, Stefan K. James, Tatevik G. Lakic, Richard C. Becker, Christopher P. Cannon, Philippe G. Steg, Anders Himmelmann, Hugo A. Katus, Robert F. Storey, Lars Wallentin, W. Douglas Weaver, Agneta Siegbahn, on behalf of the PLATO Investigators

**Affiliations:** ^1^ Department of Medical Sciences, Cardiology Uppsala University Uppsala Sweden; ^2^ Uppsala Clinical Research Center Uppsala University Uppsala Sweden; ^3^ Division of Cardiovascular Health and Disease, Heart, Lung, and Vascular Institute University of Cincinnati College of Medicine Cincinnati Ohio; ^4^ Cardiovascular Division, Brigham and Women's Hospital Boston Massachusetts; ^5^ Département Hospitalo‐Universitaire FIRE, AP‐ Paris France; ^6^ Paris Diderot University Paris France; ^7^ NHLI Imperial College ICMS, Royal Brompton Hospital London UK; ^8^ FACT (French Alliance for Cardiovascular Trials), an F‐CRIN network Paris France; ^9^ AstraZeneca Research and Development Gothenburg Sweden; ^10^ Medizinishe Klinik, Universitätsklinikum Heidelberg Heidelberg Germany; ^11^ Department of Infection, Immunity and Cardiovascular Disease University of Sheffield Sheffield UK; ^12^ Henry Ford Heart and Vascular Institute Detroit Michigan; ^13^ Department of Medical Sciences, Clinical Chemistry Uppsala University Uppsala Sweden

**Keywords:** acute coronary syndrome, inflammation, morbidity, mortality, myocardial infarction

## Abstract

**Background:**

We aimed to assess associations between circulating IL‐18 concentrations and cardiovascular outcomes in patients with acute coronary syndromes (ACS).

**Hypothesis and Methods:**

Plasma IL‐18 concentrations were measured at admission, discharge, 1 month, and 6 months in patients with ACS in the PLATelet inhibition and patient Outcomes (PLATO) trial. Associations with outcomes were evaluated with Cox regression models on the composite of CV death, spontaneous myocardial infarction (sMI), or stroke; and on CV death or sMI separately, including adjustment for clinical risk factors and biomarkers (cTnT‐hs, NT‐proBNP, cystatin C, CRP‐hs, and GDF‐15).

**Results:**

Median IL‐18 concentrations at baseline, discharge, 1 month, and 6 months were 237, 283, 305, and 320 ng/L (n = 16 636). Male sex, obesity, diabetes, and plasma levels of cystatin C, GDF‐15, and CRP‐hs were independently associated with higher IL‐18 levels. Higher baseline IL‐18 levels were associated with the composite endpoint and with CV death (hazard ratio [HR] 1.05, 95% confidence interval [95% CI] 1.02‐1.07 and HR 1.10, 95% CI 1.06‐1.14, respectively, per 25% increase of IL‐18 levels). Associations remained significant after adjustment for clinical variables but became non‐significant after adjustment for all biomarkers (HR 1.01, 95% CI 0.98‐1.04 and HR 1.04, 95% CI 1.00‐1.08, respectively). There were no associations with sMI.

**Conclusions:**

In ACS patients, IL‐18 concentrations increased after the acute event and remained increased for 6 months. Baseline IL‐18 levels were significantly associated with CV mortality, independent of clinical characteristics and indicators of renal/cardiac dysfunction but this association was attenuated after adjustment for multiple biomarkers.

## 1 INTRODUCTION

Inflammation is important for the development of atherosclerosis and inflammatory mediators synergistically amplify traditional risk factors of cardiovascular (CV) disease.^1^ An increased inflammatory activity is associated with progression of stable coronary disease but also the future risk of acute coronary syndromes (ACS).^1^, ^2^, ^3^ The role of inflammatory mediators in ACS is however less understood and routine measurement of inflammatory markers is not supported by international guidelines.^4^

Interleukin‐18 (IL‐18), a member of the IL‐1 cytokine superfamily, is a pleiotropic inflammatory cytokine expressed in several cells including macrophages and endothelial cells.^5^ Increased circulating IL‐18 concentrations have been associated with coronary artery disease and an increased risk for cardiovascular (CV) morbidity and mortality.^6^, ^7^ IL‐18 has been detected in atherosclerotic plaques and has been proposed to destabilize coronary plaques leading to subsequent thrombotic events.^3^ Nonetheless, the importance of circulating IL‐18 in patients with ACS is not well documented.^8^

The PLATelet inhibition and patient Outcomes (PLATO) trial included patients with ST‐elevation and non‐ST‐elevation ACS, and showed that ticagrelor was superior to clopidogrel treatment in preventing CV mortality, myocardial infarction (MI), or stroke.^9^, ^10^ In the current biomarker substudy, we aimed to (a) evaluate the association between IL‐18 concentrations and CV outcomes and bleeding and (b) explore the IL‐18 concentrations during follow‐up, and (c) assess possible interactions with randomized treatment (ticagrelor or clopidogrel).

## 2 MATERIALS AND METHODS

### 2.1 Design and study population

The global, randomized, placebo‐controlled PLATO trial enrolled 18 624 patients with either ST‐elevation ACS or non‐ST‐elevation ACS (Clinical Trial Registration: http://ClinicalTrials.gov NCT00391872).^9^, ^10^ The enrolled patients received optimal medical therapy including aspirin, and optional invasive strategy, and were randomized to either clopidogrel or ticagrelor treatment.^9^, ^10^ The patients were recruited between October 2006 and July 2008 and were subject to blood sampling as a part of a predefined biomarker substudy program.^9^, ^10^ The overall aims and details of the biomarker substudy program have previously been presented.^9^ All participants provided written informed consent and the study complied with the declaration of Helsinki. The study was approved by all local Ethics Committees and Institutional Review Boards.

### 2.2 Endpoint definition

The pre‐specified primary composite endpoint of the present substudy was the combination of CV death, stroke or spontaneous MI (sMI) within 1 year of follow‐up.^11^ CV death and sMI were also separately evaluated, and secondary outcomes also included PLATO‐defined non‐CABG major bleeding.^9^ Stroke was not separately evaluated, due to few events. All endpoints in the PLATO trial were centrally adjudicated by an independent and blinded clinical event adjudication committee in order to sub classify causes of death and to subdivide types of myocardial infarctions, stroke, and bleeding events.^9^, ^11^ CV death was defined as sudden death or death with no clear attributable non‐cardiovascular cause. Spontaneous myocardial infarction was defined in accordance with the universal definition of myocardial infarction, hence a non‐procedural, non‐fatal, MI type 1.^11^ The definition of stroke has previously been described.^9^

PLATO major bleeding was defined as fatal or intracranial bleeding or requiring two units of blood transfusion or with a drop in hemoglobin of >5 g/dL.^9^

### 2.3 Sampling and laboratory analysis

Baseline venous blood samples were obtained at the time of randomization within 24 hours of admission, and prior to the administration of study medication. In addition, a predefined substudy program with serial blood sampling was conducted at selected sites in order to obtain samples from 4000 patients at discharge and after 1 month after randomization and from at least 3000 of these patients also at 6 months. Patients at these selected sites were continuously invited to participate in the substudy until it was estimated that at least 3000 patients would be available for blood sampling at 6 months. Patients with a baseline blood sample and at least one additional blood sample during follow‐up were included in the serial biomarker analyses.

The venous blood was centrifuged and plasma samples were frozen in aliquots and stored at −70°C until analyzed at the Uppsala Clinical Research Center laboratory, Uppsala, Sweden. Plasma IL‐18 levels were measured using MBL human IL‐18 ELISA (MBL Medical & Biological Laboratories Co.). High‐sensitivity cardiac troponin T (cTnT‐hs, ng/L), N‐terminal pro‐B‐type natriuretic peptide (NT‐proBNP, pmol/L), and cystatin C were determined with sandwich immunoassays on the Cobas Analytics e601 Immunoanalyzer (Roche Diagnostics, Mannheim, Germany).

The growth differentiation factor‐15 (GDF‐15, ng/L) concentration was determined with Elecsys electrochemiluminescence immunoassay on a Cobas Immunoanalyzer system (Roche Diagnostics, Rotkreuz, Switzerland).^12^

White blood cells (WBC) and high‐sensitivity C‐reactive protein (CRP‐hs) were locally analyzed at site.

### 2.4 Statistical analysis

Baseline characteristics were presented by IL‐18 strata. Categorical baseline variables were presented as frequencies and percentages and compared by quartile groups of IL‐18 using *χ* ^2^ tests. Continuous variables were presented as medians with the 25th to 75th percentiles and the comparison between quartile groups of IL‐18 used the Kruskal‐Wallis test. IL‐18 concentrations were ln transformed to obtain approximate normal distribution. Correlations between IL‐18 and other biomarkers were determined using Spearman's correlation coefficients.

Crude event rates at 1‐year were estimated by IL‐18 quartile groups. The association between IL‐18 concentrations (ln transformed), on admission, with the composite primary endpoint of CV death, sMI or stroke, and secondary endpoints of CV death alone, sMI alone and non‐CABG major bleeding were assessed by multivariable Cox proportional hazards models. Similarly, the associations between IL‐18 quartile groups were examined, with the lowest quartile group as reference.

Four multivariable Cox proportional hazard models, with the hazard per 25% increase of IL‐18 level, were used. The first model (model 1) included only randomized treatment (ticagrelor or clopidogrel). Model 2 added adjustment for baseline clinical risk factors: age, sex, body mass index (BMI), diabetes, dyslipidemia, history of heart failure, hypertension, smoking status, type of ACS, chronic kidney disease, planned invasive or non‐invasive strategy, previous cardiovascular disease; and history of myocardial infarction, percutaneous coronary intervention (PCI), coronary artery bypass grafting (CABG), non‐hemorrhagic‐stroke, and peripheral artery disease (PAD). Model 3 included all previous covariates, with further addition of ln‐transformed biomarkers of renal (cystatin C) and myocardial dysfunction (cTnT‐hs and NT‐proBNP). Model 4 included all covariates from model 1‐3 with the addition of the following ln‐transformed biomarkers: WBC, CRP‐hs, and GDF‐15.

The shape of the association between continuous IL‐18 and the hazard ratio for the endpoint of interest was evaluated using restricted cubic splines with four knots.

The predefined serial measurements of IL‐18 were analyzed by analysis of covariance, with baseline IL‐18 and treatment group as independent variables, separately at each time point. Medians, as well as geometric means, of IL‐18 at the different time points (baseline, discharge, 1 month, and 6 months) were calculated.

*P* values less than .05 were considered statistically significant results. All statistical analyses were performed with SAS 9.4 (SAS Institute, Cary, North Carolina).

## 3 RESULTS

### 3.1 IL‐18 concentrations and associations with baseline characteristics and other biomarkers

Data on baseline IL‐18 concentrations were available for 16 636 patients (89.3%), with a median (interquartile interval) of 237 ng/L (180.0‐311.0) ng/L (Table 1). The IL‐18 concentrations spanned between 12.5 and 33 180 ng/L with a mean of 276 ng/L and an SD of ±436 ng/L.

**Table 1:** Baseline characteristics and biomarkers by quartile groups of IL‐18 concentrations at baseline

Characteristic	Q1	Q2	Q3	Q4	
	<180.0 ng/L	180.0‐237.0 ng/L	237.0‐311.0 ng/L	>311.0 ng/L	*P* value
	n = 4176	n = 4174	n = 4118	n = 4168	
Age (y)	63 (55‐71)	62 (54‐71)	61 (54‐70)	61 (53‐70)	<.0001
Female	1563 (37.4%)	1211 (29.0%)	1019 (24.7%)	964 (23.1%)	<.0001
BMI kg/m^2^	26.9 (24.3‐29.7)	27.3 (24.8‐30.4)	27.7 (24.9‐30.9)	27.8 (25.1‐31.0)	<.0001
**Risk factor**					
Habitual smoker	1338 (32.0%)	1484 (35.6%)	1526 (37.1%)	1561 (37.5%)	<.0001
Hypertension	2749 (65.8%)	2670 (64.0%)	2725 (66.2%)	2734 (65.6%)	.1524
Dyslipidemia	2018 (48.3%)	2030 (48.6%)	1917 (46.6%)	1886 (45.2%)	.0056
Diabetes mellitus	866 (20.7%)	985 (23.6%)	1045 (25.4%)	1256 (30.1%)	<.0001
**Medical history**					
Angina pectoris	1844 (44.2%)	1846 (44.2%)	1804 (43.8%)	1898 (45.5%)	.4053
Myocardial infarction	825 (19.8%)	883 (21.2%)	835 (20.3%)	895 (21.5%)	.1906
Congestive heart failure	215 (5.1%)	243 (5.8%)	218 (5.3%)	288 (6.9%)	.0022
PCI	539 (12.9%)	579 (13.9%)	563 (13.7%)	528 (12.7%)	.2985
CABG	265 (6.3%)	239 (5.7%)	224 (5.4%)	251 (6.0%)	.3328
TIA	107 (2.6%)	121 (2.9%)	95 (2.3%)	129 (3.1%)	.1232
Non‐hemorrhagic stroke	141 (3.4%)	159 (3.8%)	165 (4.0%)	165 (4.0%)	.4195
Peripheral arterial disease	193 (4.6%)	244 (5.8%)	275 (6.7%)	310 (7.4%)	<.0001
Chronic renal disease	133 (3.2%)	157 (3.8%)	181 (4.4%)	220 (5.3%)	<.0001
**Type of ACS**					
ST‐elevation MI	1630 (39.0%)	1648 (39.5%)	1708 (41.5%)	1733 (41.6%)	.0281
**Biomarker median (Q1‐Q3)**					
cTnT‐hs ng/L	183.0 (43.3‐627.0)	189.0 (45.4‐636.0)	188.0 (45.0‐657.0)	205.0 (48.5‐714.0)	.0369
NT‐proBNP pmol/L	61.9 (20.4‐188.1)	55.0 (18.3‐169.8)	56.7 (18.4‐176.5)	63.3 (19.5‐206.1)	.0012
GDF‐15 pg/L	1411 (1061‐1986)	1492 (1121‐2099)	1574 (1159‐2283)	1731 (1265‐2564)	<.0001
Cystatin C mg/L	0.76 (0.63‐0.92)	0.81 (0.67‐0.97)	0.84 (0.69‐1.03)	0.90 (0.73‐1.13)	<.0001
White blood cells	8.80 (7.00‐11.10)	9.10 (7.30‐11.50)	9.40 (7.50‐11.50)	9.30 (7.50‐11.90)	<.0001
CRP mg/L	2.9 (1.2‐6.9)	3.5 (1.5‐8.4)	4.1 (1.8‐10.0)	5.0 (2.1‐13.0)	<.0001

*Notes*: Categorical baseline variables were presented as frequencies and percentages and compared by quartile groups of IL‐18 using *χ* ^2^ tests. Continuous baseline variables were presented as medians and 25th to 75th percentiles and compared by quartile groups of IL‐18 using Kruskal‐Wallis tests.

Baseline characteristics in association to levels of other biomarkers by IL‐18 quartile groups are presented in Table 1. Lower age, male sex, greater weight and body mass index, diabetes and decreased kidney function were all associated with increased IL‐18 concentrations (Table 1). There were also associations between increased IL‐18 levels and increased levels of NT‐proBNP, cystatin C, GDF‐15, white blood cells and the inflammatory markers CRP‐hs, IL‐6, and IL‐10. However, Spearman correlation coefficients between IL‐18 and other ln‐transformed biomarkers revealed limited associations with the highest correlation coefficients for CRP‐hs of 0.166 and for GDF‐15 of 0.164.

The variables significantly associated with increasing IL‐18 concentration at baseline were male sex, age, diabetes, BMI > 30 kg/m^2^ and increasing levels of the biomarkers NT‐proBNP, cTnT‐hs, CRP‐hs, and cystatin C (Table 1).

### 3.2 Associations with outcomes

**Figure 1:**
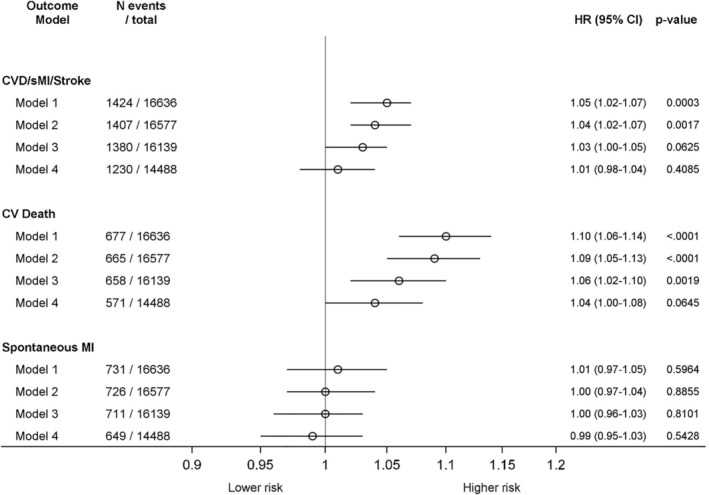
Forest plot of the hazard ratios and 95% CIs, per 25% increase in baseline IL‐18 level, for the composite endpoint, and for CV death and spontaneous MI separately during up to 12 months of follow‐up

The primary endpoint, the combination of CV death, stroke or sMI, was observed in 1424 (8.6%) patients. Crude event rates, by quartile groups of IL‐18 concentration, for the composite endpoint were 7.9%, 8.3%, 8.4%, and 9.6% (Table 2, Figure 1). The unadjusted hazard ratio (HR), per 25% increase in IL‐18 concentration, was HR 1.05 (95% CI 1.02‐1.07). After the addition of clinical variables and biomarkers, the multivariable adjusted HR was 1.01 (95% CI 0.98‐1.04) (Table 2, Figure 1).

**Table 2:** Multivariable Cox regression analysis, and event rates, on baseline IL‐18 (ng/L) and the primary composite outcome; CV death, sMI or stroke, during up to 1 year of follow‐up (n = 16 636)

Model	IL‐18 level	No of events	No of patients	Event rate (%)	HR (95% CI)	*P* value
Model 1a	Continuous	1424	16 636	8.6	1.05 (1.02‐1.07)	.0003
Model 1b	<Q1: <180.0	331	4176	7.9		.0179
	Q1‐<Q2: 180.0‐237.0	346	4174	8.3	1.05 (0.91‐1.23)	
	Q2‐<Q3: 237.0‐311.0	346	4118	8.4	1.08 (0.93‐1.25)	
	≥Q3: ≥ 311.0	401	4168	9.6	1.25 (1.08‐1.44)	
Model 2a	Continuous	1407	16 577	8.5	1.04 (1.02‐1.07)	.0017
Model 2b	<Q1: <180.0	326	4161	7.8		.0763
	Q1‐<Q2: 180.0‐237.0	343	4162	8.2	1.06 (0.91‐1.24)	
	Q2‐<Q3: 237.0‐311.0	343	4102	8.4	1.11 (0.95‐1.30)	
	≥Q3: ≥311.0	395	4152	9.5	1.21 (1.04‐1.41)	
Model 3a	Continuous	1380	16 139	8.6	1.03 (1.00‐1.05)	.0625
Model 3b	<Q1: <180.0	313	4052	7.7		.6527
	Q1‐<Q2: 180.0‐237.0	338	4057	8.3	1.07 (0.92‐1.25)	
	Q2‐<Q3: 237.0‐311.0	338	4008	8.4	1.07 (0.92‐1.25)	
	≥Q3: ≥311.0	391	4022	9.7	1.10 (0.95‐1.29)	
Model 4a	Continuous	1230	14 488	8.5	1.01 (0.98‐1.04)	.4085
Model 4b	<Q1: <180.0	280	3660	7.7		.9427
	Q1‐<Q2: 180.0‐237.0	308	3685	8.4	1.05 (0.89‐1.24)	
	Q2‐<Q3: 237.0‐311.0	302	3598	8.4	1.02 (0.86‐1.20)	
	≥Q3: ≥311.0	340	3545	9.6	1.03 (0.87‐1.21)	

*Notes*: Model 1 includes ln(IL‐18) and randomized treatment. Model 2 includes ln(IL‐18) and clinical variables. Model 3 includes the covariates from Model 2 and biomarkers: ln(cystatin C), ln(cTnT‐hs), ln(NT‐proBNP). Model 4 includes the covariates from Model 3 and biomarkers: ln(CRP), ln(GDF‐15), and ln(WBC).

Event rates for CV death alone per increasing quartile groups of IL‐18 were 3.2%, 3.4%, 4.4%, and 5.3% (Table 3). Multivariable adjusted HR per 25% increase in IL‐18 concentration on CV death was significantly associated with outcome, even after the adjustment for randomized treatment, clinical variables and the biomarkers cystatin C, cTnT‐hs, and NT‐proBNP. However, after additional adjustment with the biomarkers WBC, GDF‐15 and CRP‐hs in model 4, the association between IL‐18 and CV death was insignificant (Table 3, Figure 1).

**Table 3:** Multivariable Cox regression analysis, and event rates, on baseline IL‐18 (ng/L) and CV death during up to 1 year of follow‐up (n = 16 636)^a^

Model	IL‐18 level	No of events	No of patients	Crude event rate (%)	HR (95% CI)	*P* value
Model 1a	Continuous	677	16 636	4.1	1.10 (1.06‐1.14)	<.0001
Model 1b	<Q1: <180.0	132	4176	3.2		<.0001
	Q1‐<Q2: 180.0‐237.0	143	4174	3.4	1.09 (0.86‐1.38)	
	Q2‐<Q3: 237.0‐311.0	180	4118	4.4	1.41 (1.12‐1.76)	
	≥Q3: ≥311.0	222	4168	5.3	1.73 (1.40‐2.15)	
Model 2a	Continuous	665	16 577	4.0	1.09 (1.05‐1.13)	<.0001
Model 2b	<Q1: <180.0	130	4161	3.1		<.0001
	Q1‐<Q2: 180.0‐237.0	140	4162	3.4	1.09 (0.86‐1.39)	
	Q2‐<Q3: 237.0‐311.0	177	4102	4.3	1.45 (1.15‐1.82)	
	≥Q3: ≥311.0	218	4152	5.3	1.63 (1.31‐2.04)	
Model 3a	Continuous	658	16 139	4.1	1.06 (1.02‐1.10)	.0019
Model 3b	<Q1: <180.0	128	4052	3.2		.0169
	Q1‐<Q2: 180.0‐237.0	138	4057	3.4	1.07 (0.84‐1.36)	
	Q2‐<Q3: 237.0‐311.0	175	4008	4.4	1.32 (1.05‐1.67)	
	≥Q3: ≥311.0	217	4022	5.4	1.36 (1.08‐1.71)	
Model 4a	Continuous	571	14 488	3.9	1.04 (1.00‐1.08)	.0645
Model 4b	<Q1: <180.0	112	3660	3.1		.2917
	Q1‐<Q2: 180.0‐237.0	123	3685	3.3	1.02 (0.79‐1.32)	
	Q2‐<Q3: 237.0‐311.0	152	3598	4.2	1.20 (0.94‐1.54)	
	≥Q3: ≥311.0	184	3545	5.2	1.19 (0.93‐1.52)	

*Notes*: Model 1 includes ln(IL‐18) and randomized treatment. Model 2 includes ln(IL‐18) and clinical variables. Model 3 includes the covariates from Model 2 and biomarkers: ln(cystatin C), ln(cTnT‐hs), ln(NT‐proBNP). Model 4 includes the covariates from Model 3 and biomarkers: ln(CRP), ln(GDF‐15), and ln(WBC).

a Quartile groups IL‐18: Q1; <180, Q2; 181‐236, Q3; 237‐311, Q4; 312.

The crude event rates for sMI (n = 731) alone per increasing quartile groups of IL‐18 were 4.5%, 4.7%, 4.0%, and 4.4%. There was no association between the IL‐18 level and sMI: HR 0.99 (95% CI 0.95‐1.03) (Figure 1). Neither was there any relationship between non‐CABG major bleeding (n = 602) and increasing quartile group of IL‐18 levels: 3.8%, 3.6%, 3.4%, and 3.6%, respectively; HR 0.96 (95% CI 0.91‐1.00) (*P* value = 0.06).

### 3.3 Interaction between IL‐18 and the effects of randomized treatment on outcome

There was no significant interaction between the IL‐18 quartile groups and the effects on outcomes by the randomized treatment. The associations between IL‐18 levels and the effects of randomized treatment (ticagrelor vs clopidogrel) were also investigated by restricted cubic splines (Figure S1A, B, C and D). There were similar associations between IL‐18 levels and outcomes irrespective of treatment with ticagrelor or clopidogrel, except for sMI where there was a trend for increasing events with increasing IL‐18 level in the clopidogrel group but not the ticagrelor group.

### 3.4 IL‐18 concentrations and variables associated with IL‐18 concentrations during follow‐up

Patients with a baseline IL‐18 sample and at least one subsequent follow‐up sample (at discharge, 1 month, or 6 months) were included in the serial biomarker subset (n = 4583) (Table 4). There was a significant increase in IL‐18 concentrations during the first month, with a similar level at 1 and 6 months after the index event in ACS survivors (Table 4). The increase in IL‐18 concentrations was observed irrespective of randomized treatment (Table 4).

**Table 4:** Median IL‐18 concentrations (ng/L) during follow‐up: at baseline, discharge and after 1 and 6 months^a^

Visit	Overall			Ticagrelor		Clopidogrel		
	N	Median IL‐18 (ng/L; Q1‐Q3)	*P* value	N	Median IL‐18 (ng/L; Q1;Q3)	N	Median IL‐18 (ng/L; Q1;Q3)	*P* value (rand. treatment)
**Baseline**	4585	233 (179‐306)		2277	232 (179‐306)	2306	233 (179‐305)	
**Discharge**	4461	284 (216‐369)		2211	284 (215‐367)	2245	283 (217‐370)	
Change from baseline	4461	46 (2‐97)	<.0001	2211	46 (1‐95)	2245	47 (4‐99)	.2471
**1 month**	3982	306 (233‐395)		1982	304 (232‐395)	2000	308 (234‐396)	
Change from baseline	3982	72 (17‐132)	<.0001	1982	68 (15‐129)	2000	76 (18‐135)	.1255
Change from discharge	3859	25 (−34‐84)	<.0001					
**6 months**	1929	320 (244‐414)		937	323 (245‐414)	992	318 (243‐413)	
Change from baseline	1929	80 (28‐142)	<.0001	937	79 (26‐141)	992	81 (30‐144)	.7980
Change from discharge	1856	35 (−23‐96)	<.0001					
Change from 1 month	1907	17 (−43‐80)	<.0001					

a To the left presented overall and to the right in strata by randomized treatment. All subjects with a baseline value and at least one follow‐up sample were included in the analysis (n = 4583). The model includes a non‐parametric test with the baseline value as a covariate.

The following variables were significantly associated with the IL‐18 concentration at three out of the four occasions during follow‐up: male sex, obesity, diabetes and biomarkers of renal function (cystatin C), and inflammation (WBC, CRP‐hs, GDF‐15, Il‐10) (Table S1).

## 4 DISCUSSION

The main findings of the current study were that, in patients with ACS, the IL‐18 concentration increased during 1 month after the acute event and remained at around the same level for at least 6 months. Higher IL‐18 levels were consistently associated with male sex, higher BMI, diabetes, decreased renal function, and other inflammatory biomarkers. The IL‐18 level at baseline was significantly associated with CV mortality independent of clinical characteristics and indicators of renal and cardiac dysfunction. However, the association with CV mortality was attenuated and did not remain statistically significant after further adjustment with biomarkers, including GDF‐15 and inflammatory markers. Finally, there was no association between IL‐18 level and the risk of myocardial infarction.

Inflammation is an important factor for the development of atherosclerosis, coronary plaques, and plaque rupture.^1^, ^13^, ^14^ A large number of inflammatory markers have been described, and IL‐18 has been reported associated with both presence of CV disease and clinical outcomes.^6^, ^7^ However, the exact role of these inflammatory mediators in ACS is complex and the importance of IL‐18 is only partly understood. The predictive accuracy of these markers can be influenced by the clinical context and the sample time, which is not always well recorded, and consequently the levels of an inflammatory marker prior to an event, including IL‐18, is difficult to estimate.^14^, ^15^ As might be expected, in the current study of patients with ACS, levels of IL‐18 increased from the first measurement at baseline to discharge.^16^ Further, the concentrations remained elevated from 1 month until the last observation at 6 months. These findings suggest that the inflammatory response by IL‐18 to ACS is rapid but the possible return to pre‐ACS levels is slow, as if or when it returns to baseline is currently unknown. In comparison, CRP‐hs and WBC have a much faster regression, tapering within days.

The prognostic impact of IL‐18 has previously been examined in a smaller cohort (n = 1261) of patients with ACS, which also showed a significant association with long‐term (10 years) overall mortality but not with MI.^8^ In our study, comprising a more than 10 times larger cohort of patients with ACS, the overall mortality was 5.6% of which the vast majority was CV deaths. An association between the IL‐18 levels and CV mortality was also observed and, importantly, the association between IL‐18 and CV death remained significant even after the adjustment for important CV risk markers as cystatin C, NT‐proBNP, and cTnT‐hs.

But after the addition of inflammatory biomarkers, this association was no longer statistically significant. This clearly indicates that inflammatory activity is a contributing factor to fatal CV events beyond the risk associated with clinical variables and cardiorenal biomarkers. The attenuation of the association after the adjustment also for other inflammatory markers like CRP‐hs, WBC, and CV risk marker GDF‐15 may hypothesize that these markers share common pathways of the underlying inflammation in patients with ACS. Thus, although the prognostic importance of IL‐18 is limited if other inflammatory biomarkers are available, IL‐18 may still indicate important underlying processes contributing to fatal outcomes in ACS. Additionally, one should not rule out that a biomarker, still associated with outcome after adjustment for important biomarkers cystatin C, NT‐proBNP, and cTnT‐hs, may provide important information in certain subgroups or cohorts.^17^

The biological function of IL‐18 includes the induction of several pro‐inflammatory mediators like the cytokines interferon‐gamma (IFN‐γ), IL‐1β, and IL‐6.^18^ IL‐18 has also been proposed to activate circulating monocytes that eventually can transform into foam cells, the hallmark cell in atherosclerosis.^1^, ^13^ The production of IL‐18 is regulated by the inflammasome, a complex of intracellular signal proteins, that modifies the inflammatory response.^18^, ^19^ Human macrophages and endothelial cells express IL‐18 receptors and activation of these receptors induce the production of mediators with defined roles in thrombosis and atherogenesis, including IL‐6 and intracellular adhesion molecule (ICAM)‐1.^5^ Consequently, the intricate activation and multiple features of the pleiotropic cytokine IL‐18 can result in a broad variety of responses.^3^, ^18^ Despite the proposed role of IL‐18 in atherogenesis and thrombosis, we did not find IL‐18 concentrations to be associated with sMI in the present study. Further investigations into the underlying mechanisms for this association, for example, association to individual causes of death such as congestive heart failure or arrhythmic death, might yield additional important information.

We have previously reported that clopidogrel treatment, compared with ticagrelor treatment, was associated with a higher incidence of severe pulmonary infections or sepsis and an increased mortality.^20^ This finding was accompanied with a lower leucocyte count at one month and lower mean CRP‐hs and IL‐6 concentrations in patients randomized to clopidogrel, indicating possible effects of the randomized antiplatelet treatment on immune signaling.^20^ However, we found no evidence that the randomized antiplatelet treatment affected IL‐18 concentrations. Rather, an enhanced and sustained inflammatory response for more than 6 months after ACS was observed, irrespective of randomized antiplatelet treatment.

## 5 LIMITATIONS

The PLATO trial enrolled a broad ACS population but, as in several clinical trials, certain patient groups were excluded, for example, patients requiring dialysis or patients with recent significant bleeding. The current substudy investigated IL‐18 concentrations at baseline, and all samples were drawn within 24 h of admission, nonetheless, the time span between symptom onset and sample time is not adjusted for and may have affected the observed IL‐18 results at baseline. Further, the reported IL‐18 concentrations were circulating IL‐18, and we were not able to either measure or estimate total IL‐18 levels.

## 6 CONCLUSION

In patients with ACS, the IL‐18 concentration increased during the first month after the acute event and remained at similar level at 6 months. The IL‐18 levels were consistently associated with male sex, higher BMI, diabetes, decreased renal function, and to other inflammatory biomarkers. The IL‐18 level at baseline was significantly associated with CV‐mortality independent of clinical characteristics and indicators of renal and cardiac dysfunction. However, the association with CV mortality was attenuated and did not remain statistically significant after the adjustment for further inflammatory biomarkers and GDF‐15. Although the prognostic importance of IL‐18 is limited if other prognostic inflammatory biomarkers are available, IL‐18 may still be part of underlying inflammatory processes contributing to fatal outcomes in ACS.

## CONFLICT OF INTEREST

Axel Åkerblom received institutional research grants, speaker honoraria, and consultancy/advisory board member fees from AstraZeneca; institutional research grants from Roche Diagnostics.

Stefan K. James received institutional research grants, honoraria, and consultancy/advisory board member fees from AstraZeneca; institutional research grant and consultant/advisory board fee from Medtronic; institutional research grants and honoraria from The Medicines Company; consultancy/advisory board member fees from Janssen and Bayer.

Tatevik G. Lakic received institutional research grants from AstraZeneca and Roche Diagnostics.

Richard C. Becker received scientific advisory board member fees from AstraZeneca, Bayer, CryoLife, and Ionis Pharmaceuticals; safety reviewing committee member fees from Portola and Akcea Therapeutics.

Christopher P. Cannon received research grants from Amgen, Boehringer Ingelheim, Bristol‐Myers Squibb, Daiichi Sankyo, Janssen, and Merck; consultancy fees from Aegerion, Alnylam, Amarin, Amgen, Boehringer Ingelheim, Bristol‐Myers Squibb, Corvidia, Eisai, Innovent, Janssen, Kowa, Merck, Pfizer, Regeneron, and Sanofi.

Philippe G. Steg received research grants and speaker/consultancy fees from Merck, Sanofi, and Servier; speaker/consultancy fees from AstraZeneca, Amarin, Amgen, Bayer, Boehringer Ingelheim, Bristol‐Myers Squibb, Janssen, NovoNordisk, Novartis, Pfizer, and Regeneron.

Anders Himmelmann is an employee of AstraZeneca.

Hugo A. Katus received personal fees from AstraZeneca, Daiichi, Novartis, Bayer, Roche Diagnostics, Berlin Chimie, and NovoNordisk.

Robert F. Storey received institutional research grants/support from AstraZeneca; consultancy fees from Amgen, AstraZeneca, Bayer, Bristol Myers Squibb/Pfizer, GlyCardial, Haemonetics, Idorsia, Novartis and Thromboserin; honoraria from AstraZeneca, Bayer, Bristol Myers Squibb/Pfizer, and Medscape.

Lars Wallentin received institutional grants from AstraZeneca, Roche Diagnostics, Merck & Co, GlaxoSmithKline, Boehringer Ingelheim, and Bristol‐Myers Squibb/Pfizer; consultancy fees from Abbott; holds two patents licensed to Roche Diagnostics.

W. Douglas Weaver had nothing to report.

Agneta Siegbahn received institutional research grants from AstraZeneca, Roche Diagnostics, Bristol‐Myers‐Squibb/Pfizer, Boehringer Ingelheim, and GlaxoSmithKline; consultancy fees from Olink Proteomics.

## Supporting information


**Appendix S1.** Supporting information.
