# Supplementary material for: Interleukin‐18 in patients with acute coronary syndromes
Source: Clin Cardiol. 2019 Oct 9;42(12):1202–9. doi: 10.1002/clc.23274 (PMC6906991; doi:10.1002/clc.23274)
Supplement: Supplementary file 1 — Appendix S1. Supporting information. [file CLC-42-1202-s001.pdf]

# SUPPLEMENT

Åkerblom et al: Interleukin-18 in ACS

**Supplementary figures 1A, B, C, and D**

Restricted cubic splines of the primary endpoint (A), CV death separately (B), spontaneous MI separately (C) and major bleeding (D) during up to one year of follow up by strata of randomized treatment.

**Supplementary table**

Multivariable effect of clinical risk factors and biomarkers on the IL-18 levels at baseline, discharge, one month, and six months.

**Supplementary figure 1A - the composite endpoint of CV death, sMI, or stroke.**

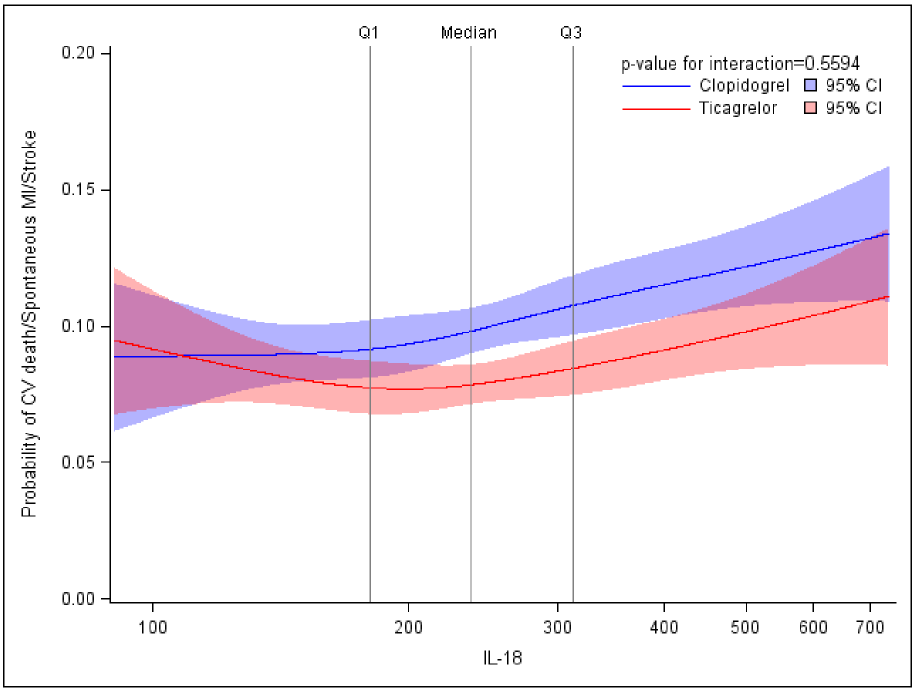


**1B - CV death separately**

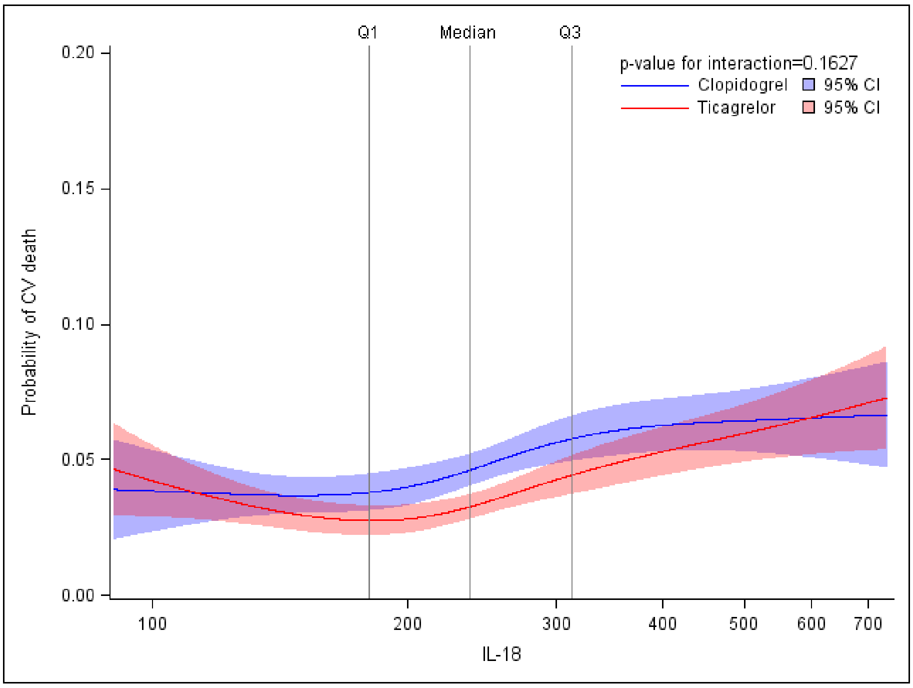

## 1C - spontaneous MI separately

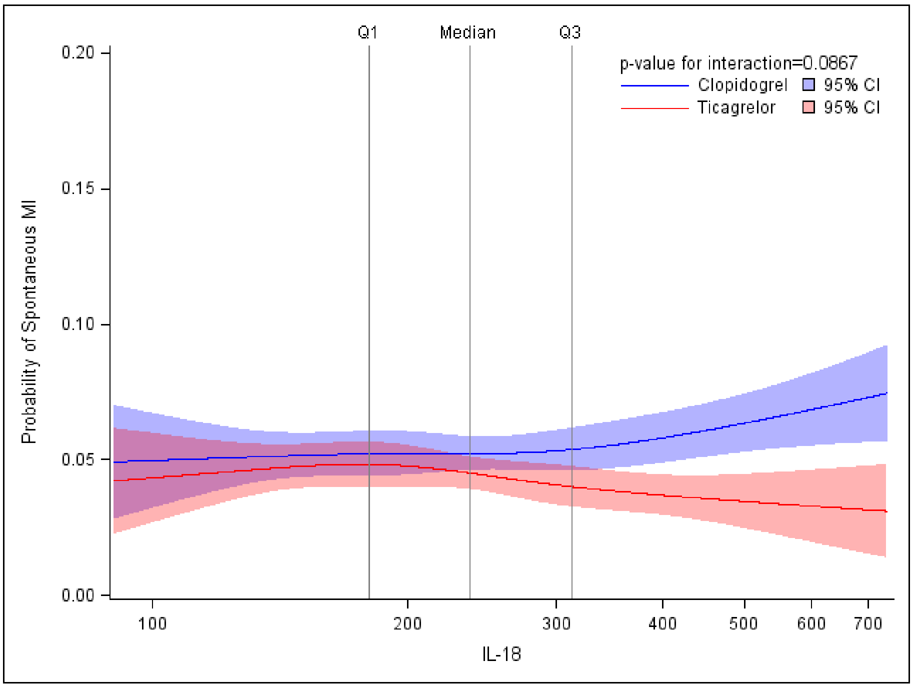


## 1D - major bleeding

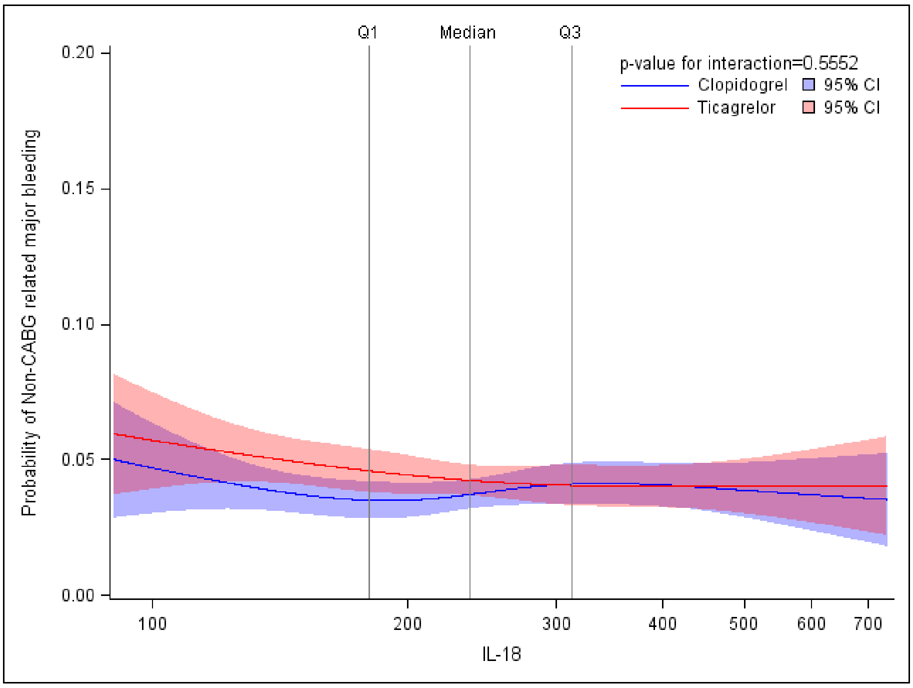

**Supplementary table**

Multivariable effect of clinical risk factors and biomarkers on the IL-18 levels at baseline, discharge, one month, and six months.

The model includes baseline variables and biomarkers, however at one month and six months the model is based upon biomarkers obtained at one month and six months, respectively.

| Visit | Baseline | | | Discharge | | | 1 month | | | 6 months | | |
|---|---|---|---|---|---|---|---|---|---|---|---|---|
| **Background characteristic** | **Rel incr** | **95% CI** | **P-value** | **Rel incr** | **95% CI** | **P-value** | **Rel incr** | **95% CI** | **P-value** | **Rel incr** | **95% CI** | **P-value** |
| Ticagrelor vs Clopidogrel | 0.997 | (0.972 - 1.022) | 0.816 | 0.978 | (0.952 - 1.005) | 0.112 | 0.999 | (0.970 - 1.029) | 0.948 | 1.004 | (0.962 - 1.047) | 0.864 |
| Age,10 year increase | 0.959 | (0.945 - 0.974) | <.0001 | 0.965 | (0.950 - 0.981) | <.0001 | 0.992 | (0.974 - 1.010) | 0.395 | 0.985 | (0.958- 1.012) | 0.261 |
| Female vs male | 0.887 | (0.861- 0.914) | <.0001 | 0.900 | (0.872 - 0.929) | <.0001 | 0.928 | (0.896 - 0.961) | <.0001 | 0.899 | (0.852 - 0.948) | <.0001 |
| BMI >= 30 kg/m2 | 1.057 | (1.026 - 1.088) | <.0001 | 1.039 | (1.007 - 1.071) | 0.018 | 1.046 | (1.012 - 1.081) | 0.008 | 1.014 | (0.965 - 1.065) | 0.580 |
| Habitual smoker | 0.990 | (0.961 - 1.021) | 0.532 | 1.035 | (1.003 - 1.069) | 0.035 | 1.005 | (0.971 - 1.041) | 0.767 | 1.012 | (0.963 - 1.064) | 0.634 |

| | | | | | | | | | | | | |
|---|---|---|---|---|---|---|---|---|---|---|---|---|
| Hypertension | 0.988 | (0.959 - 1.017) | 0.404 | 0.984 | (0.954 - 1.015) | 0.308 | 0.974 | (0.942 - 1.007) | 0.118 | 0.987 | (0.940 - 1.036) | 0.601 |
| Dyslipidemia | 0.999 | (0.972 - 1.027) | 0.961 | 0.988 | (0.959 - 1.017) | 0.414 | 1.001 | (0.970 - 1.033) | 0.949 | 0.993 | (0.948 - 1.040) | 0.759 |
| Diabetes | 1.058 | (1.024 - 1.093) | 0.001 | 1.072 | (1.035 - 1.110) | <.0001 | 1.021 | (0.983 - 1.060) | 0.292 | 1.015 | (0.960 - 1.073) | 0.593 |
| Angina pectoris | 1.027 | (0.998 - 1.057) | 0.072 | 1.016 | (0.9877 - 1.048) | 0.301 | 1.030 | (0.998 - 1.064) | 0.070 | 1.014 | (0.968 - 1.063) | 0.559 |
| Myocardial infarction | 0.997 | (0.959 - 1.036) | 0.871 | 0.972 | (0.932 - 1.012) | 0.170 | 1.015 | (0.971 - 1.061) | 0.507 | 1.012 | (0.950 - 1.078) | 0.708 |
| CHF | 1.009 | (0.951 - 1.071) | 0.772 | 1.031 | (0.969 - 1.098) | 0.333 | 1.052 | (0.982 - 1.127) | 0.146 | 1.056 | (0.955 - 1.168) | 0.287 |
| PCI | 0.986 | (0.942 - 1.034) | 0.565 | 1.026 | (0.977 - 1.078) | 0.312 | 0.974 | (0.923 - 1.027) | 0.324 | 0.997 | (0.925 - 1.075) | 0.936 |
| CABG | 0.970 | (0.910 - 1.034) | 0.355 | 1.028 | (0.961 - 1.100) | 0.428 | 1.003 | (0.932 - 1.079) | 0.940 | 0.939 | (0.843 - 1.045) | 0.245 |
| TIA | 0.912 | (0.832 - 1.000) | 0.050 | 0.934 | (0.848 - 1.029) | 0.165 | 0.919 | (0.828 - 1.022) | 0.118 | 0.876 | (0.755 - 1.015) | 0.078 |

| | | | | | | | | | | | | |
|---|---|---|---|---|---|---|---|---|---|---|---|---|
| Non-haemorrhagic stroke | 0.977 | (0.910 - 1.049) | 0.517 | 0.972 | (0.901 - 1.049) | 0.466 | 1.074 | (0.987 - 1.168) | 0.097 | 1.026 | (0.909 - 1.159) | 0.673 |
| PAD | 0.996 | (0.944 - 1.050) | 0.870 | 1.002 | (0.946 - 1.060) | 0.956 | 0.983 | (0.926 - 1.044) | 0.586 | 1.037 | (0.950 - 1.132) | 0.416 |
| Chronic renal disease | 0.989 | (0.917 - 1.067) | 0.773 | 0.977 | (0.901 - 1.059) | 0.568 | 0.970 | (0.888 - 1.059) | 0.492 | 1.066 | (0.936 - 1.214) | 0.336 |
| STEMI* | 1.012 | (0.983 - 1.042) | 0.419 | 1.001 | (0.970 - 1.034) | 0.934 | 1.004 | (0.972 - 1.037) | 0.805 | 1.042 | (0.996 - 1.090) | 0.076 |
| Troponin (cTn-hs),10% increase | 1.000 | (0.999 - 1.001) | 0.779 | 0.999 | (0.998 - 1.000) | 0.008 | 1.000 | (0.997 - 1.002) | 0.737 | 1.002 | (0.998 - 1.006) | 0.467 |
| NT-proBNP,10% increase | 0.999 | (0.998 - 1.000) | 0.038 | 0.998 | (0.997 - 1.000) | 0.011 | 0.998 | (0.996 - 1.000) | 0.012 | 0.998 | (0.995 - 1.000) | 0.021 |
| GDF-15,10% increase | 1.009 | (1.006 - 1.012) | <.0001 | 1.006 | (1.002 - 1.009) | 0.002 | 1.005 | (1.001 - 1.009) | 0.027 | 1.002 | (0.996 - 1.009) | 0.438 |
| Cystatin-C.10% increase | 1.022 | (1.017 - 1.027) | <.0001 | 1.029 | (1.024 - 1.035) | <.0001 | 1.025 | (1.019 - 1.031) | <.0001 | 1.030 | (1.020 - 1.040) | <.0001 |

| | | | | | | | | | | | | |
|---|---|---|---|---|---|---|---|---|---|---|---|---|
| WBC.10% increase | 0.997 | (0.992 - 1.001) | 0.120 | N/A | N/A | N/A | 1.001 | (0.995 - 1.007) | 0.837 | 1.001 | (0.993 - 1.0100) | 0.766 |
| CRP.10% increase | 1.002 | (1.001 - 1.003) | <.0001 | 1.002 | (1.001 - 1.004) | 0.006 | 1.004 | (1.003 - 1.006) | <.0001 | 1.004 | (1.002 - 1.006) | 0.000 |
| IL-6.10% increase | 0.999 | (0.997 - 1.001) | 0.454 | 1.001 | (0.999 - 1.003) | 0.439 | 1.000 | (0.998 - 1.003) | 0.819 | 1.003 | (0.999 - 1.006) | 0.163 |
| IL-10.10% increase | 1.001 | (1.000 - 1.001) | 0.140 | 1.002 | (1.001 - 1.003) | <.0001 | 1.002 | (1.001 - 1.003) | <.0001 | 1.002 | (1.000 - 1.003) | 0.017 |

Linear model for ln-transformed IL-18 at baseline. discharge. 1-month and 6-months. The relative increase is the adjusted geometric mean ratio between subgroups or for the stated change in continuous variables

* STEMI at admission
